# A Qualitative Needs Analysis of Skin Cancer Care from the Perspectives of Patients, Physicians, and Health Insurance Representatives—A Case Study from Eastern Saxony, Germany

**DOI:** 10.3390/curroncol29040212

**Published:** 2022-04-09

**Authors:** Josephine Mathiebe, Lydia Reinhardt, Maike Bergmann, Marina Lindauer, Alina Herrmann, Cristin Strasser, Friedegund Meier, Jochen Schmitt

**Affiliations:** 1 Center for Evidence-Based Healthcare, University Hospital Carl Gustav Carus and Carl Gustav Carus Faculty of Medicine, Technische Universität Dresden, 01307 Dresden, Germany; alina.herrmann@uniklinikum-dresden.de (A.H.); jochen.schmitt@uniklinikum-dresden.de (J.S.); 2 Skin Cancer Center at the University Cancer Center and National Center for Tumor Diseases, Department of Dermatology, University Hospital Carl Gustav Carus and Carl Gustav Carus Faculty of Medicine, Technische Universität Dresden, 01307 Dresden, Germany; lydia.reinhardt@uniklinikum-dresden.de (L.R.); maike.bergmann@uniklinikum-dresden.de (M.B.); cristin.strasser@uniklinikum-dresden.de (C.S.); friedegund.meier@uniklinikum-dresden.de (F.M.); 3 Study Office Medical Oncology, Medical Department I, Carl Gustav Carus Faculty of Medicine, Technische Universität Dresden, 01307 Dresden, Germany; marina.lindauer@uniklinikum-dresden.de

**Keywords:** skin cancer, melanoma, intersectoral collaboration, qualitative research, regional care network, rural health service

## Abstract

Skin cancer is one of the most common cancers worldwide and the number of patients is steadily increasing. In skin cancer care, greater interdisciplinary cooperation is required for prevention, early detection, and new complex systemic therapies. However, the implementation of innovative medical care is a major challenge, especially for rural regions with an older than average, multimorbid population, with limited mobility, that are long distances from medical facilities. Solutions are necessary to ensure comprehensive oncological care in rural regions. The aim of this study was to identify indicators to establish a regional care network for integrated skin cancer care. To capture the perspectives of different stakeholder groups, we conducted two focus groups with twenty skin cancer patients and their relatives, a workshop with eight physicians, and three semi-structured interviews with health insurance company representatives. Qualitative data were recorded, transcribed, and analyzed following Mayring’s content analysis methods. We generated ten categories based on the reported optimization potentials; five categories were assigned to all three stakeholder groups: Prevention and early diagnosis, accessibility of physicians/clinics, physicians’ resources, care provider’s responsibilities, and information exchange. The results indicate the need for stronger integration of care in the region. They provide the basis for regional networking as, for example, the conception of treatment pathways or telemedicine with the aim to improve a comprehensive skin cancer care. Our study should raise awareness and postulate as a demand that all patients receive guideline-based therapy, regardless of where they live.

## 1. Introduction

Skin cancer is one of the most common cancers, with an incidence that has been increasing for decades [1]. Non-melanoma skin cancer ranks fifth worldwide, but is the most frequently diagnosed cancer in Germany [2,3]. Malignant melanoma occurs less frequently, but metastasizes much more frequently; thus, it has a less favorable prognosis. If melanoma is diagnosed and treated at an early stage, the prognosis is much more favorable [4]. New treatment options such as immune checkpoint inhibitors and targeted therapies have led to improved overall survival in skin cancer [5,6,7,8]. However, successful therapies require a high level of physician competence, patient compliance, interdisciplinary treatment, and side effect management. Both the rising incidence and the increasingly complex treatment of patients with advanced or metastasized skin tumors thus represent a major challenge. Early diagnosis and guideline-based medical care for skin cancer patients should therefore be offered in a coordinated manner across sectors, regardless of place of residence, age, gender, and origin [9,10].

Taking the example of Germany, two nationwide evaluations of cancer registry data show that the opportunity of an initial diagnosis of malignant melanoma at an early stage is unequally distributed. In Western Germany, this opportunity is significantly higher compared to Eastern Germany [11]. In addition, the mortality risk for advanced malignant melanoma also varies from region to region, with patients in Eastern Germany having a 47% higher risk of death compared to patients with melanoma in Western Germany [12]. The provision of medical care for skin cancer patients, especially melanoma patients, is complex, with different specialities and disciplines being involved such as dermatologists, oncologists, and general practitioners in the outpatient sector, and general care hospitals and specialized cancer centers in urban areas. The cooperation and regional networking of hospitals and practices are essential to ensure individualized and effective therapy solutions for all patients [13,14]. In rural regions, however, it can be assumed that physicians and patients will be confronted with supply problems. This involves, among other things, long journeys to the nearest practice or hospital, as well as a generally elderly, multimorbid population with limited mobility and high medical needs.

Since 2011, the availability of new, effective, but also toxic, drug-based tumor therapies for melanoma and non-melanoma skin cancer has increased. For the success of the therapy, multiple aspects are relevant: the knowledge of new therapies, side effect management, interdisciplinary therapy, and patient competence. These are not only important in comprehensive cancer centers, but also particularly in a local presence close to the patients. For the early detection of skin cancer, physician competence and patient awareness are also relevant.

To cope with these challenges in healthcare provision for patients with skin cancer, regional care networks have been suggested to improve access to high-level clinical care, irrespective of the place of residence of patients. Various networks and integrated forms of care worldwide are dedicated to the care of patients in rural areas or tumor patients [15,16,17,18,19]. Therefore, the Center for Evidence-Based Healthcare and the Skin Cancer Center at the Technical University Dresden conducted a needs analysis considering the different perspectives of patients, physicians, and health insurance company representatives. Our aim was to assess the general acceptance of a regional network for the integrated care of skin cancer patients and to obtain starting points for its design. We wanted to know whether the patients, physicians, and health insurance representatives who live in the region or work for the region also consider the abovementioned needs to be relevant, and whether there is interest in their improvement.

Here, we present the methodological design and results of our qualitative needs analysis. We focus on the extent to which the participants see potential for improvement in the healthcare provision of skin cancer patients in the study region. We also report on the experience we have gathered during the project and derive recommendations for the design of a network.

## 2. Materials and Methods

### 2.1. Study Design and Setting

We used a qualitative study design to explore the perspectives of different stakeholder groups on integrated skin cancer care. The study region of Eastern Saxony is characterized by larger rural areas in the east, with approximately 552,000 inhabitants and the city of Dresden with about 561,000 inhabitants [20]. The melanoma incidence per 100,000 citizens in Saxony is 16.1 for men and 12.1 for women (years 2014–2015) [21]. This mainly affects adults over the age of 75. Specifically for Eastern Saxony, the proportion of over 65 year olds is over 26% [22]. In Dresden, there are two Comprehensive Cancer Centers that provide specialized skin cancer care for the region [23,24].

Patients were interviewed in focus groups because this methodology allows for group interaction and can thus increase diversity of opinion [25,26]. The capture of physicians’ needs was conducted in a workshop to (a) gather many opinions and (b) develop proposals for a possible regional network. Due to the competitive situation between the health insurance providers, their needs were collected individually with semi-structured interviews (face-to-face) [25]. For reporting our qualitative research, we used the COREQ qualitative research reporting checklist [27] (Supplementary Materials Table S1). To self-evaluate our study quality, we used the CASP (Critical Appraisal Skills Programme) Qualitative Studies Checklist [28] (Supplementary Materials Table S2).

### 2.2. Sampling

#### 2.2.1. Focus Groups with Patients

Inclusion criteria were established for patient selection: (a) diagnosis of malignant melanoma (we invited only melanoma patients, as they usually have a more severe and complex disease than patients with non-melanoma skin cancer), (b) current/past treatment in the study region, and (c) health and basic cognitive ability to participate in the study, according to the patient’s medical history. Potential study participants were identified via the Skin Cancer Center of the University Hospital Dresden and invited to participate in the study by mail (n = 83). A total of 46 patients indicated a preferred date to participate in a focus group. Due to the explorative approach, as well as research economic reasons, two focus groups (with 9 and 11 participants each) were conducted. In order to capture as many perspectives as possible and to obtain a heterogeneous sample, sampling was performed using the maximum variation method [29,30]. For this purpose, patients with different characteristics in terms of age, gender, stage of disease, and size of the place of residence were selected (Table 1). The size of the place of residence was defined according to the Federal Institute for Research on Building, Urban Affairs and Spatial Development [31], as indicated in Table 1. At the request of the patients, relatives could participate as proxies or companions. A travel allowance of EUR 50 was reimbursed per patient or proxy.

#### 2.2.2. Workshop with Physicians

For the workshop, only primary, secondary, and tertiary care physicians with practical experience in the diagnosis and treatment of patients with skin cancer working in Eastern Saxony were included. The sampling procedure was multi-stage. First, the most frequent referring physicians of the Skin Cancer Center of the University Hospital Dresden (dermatologists in outpatient practices (n = 33), general practitioners (n = 12), internists/oncologists in outpatient practices (n = 8), and clinicians (n = 5)) were informed about the study by mail and invited to the workshop. In addition to the most frequent referring physicians, further physicians were invited to the workshop in a second step (dermatologists in outpatient practices from the study region (n = 17) and clinicians from the field of dermatooncology researched online (n = 5)). Analogous to that for patients, two workshops were planned, each with 10 physicians. Due to the low number of registrations (n = 16) and limited time resources of the physicians, a workshop with 8 participants was finally organized. For this purpose, (a) the date was chosen from all registrations, on which most physicians of different disciplines and places of practice agreed by vote, and (b) a venue (Bautzen) was determined, which also seemed to be easily accessible for participants outside the city of Dresden. Study participants were offered a travel allowance of EUR 50.

#### 2.2.3. Semi-Structured Interviews with Health Insurance Company Representatives

For the semi-structured interviews with health insurance company representatives, a search was conducted for statutory health insurance companies in Germany with a large number of insured persons, from which a high number of insured persons with skin cancer was determined. At the same time, a search was conducted for private health insurance companies in Germany, as their perspective was also to be considered within the framework of the study. The representatives researched were informed about the study by mail, e-mail, or telephone, and invited to participate in an interview. The interviewer arranged individual appointments with the health insurance company representatives. Our study focused mainly on the patient´s perspective. However, we also wanted to engage the other stakeholders involved in skin cancer care to gain insights into their views as well. When planning the sample size, we focused on purposive selection of different stakeholders rather than on the size of the individual groups. Thus, a total of 31 subjects participated in the study.

### 2.3. Survey Instruments

Interview and moderation guidelines with openly formulated guiding questions were developed in advance (Supplementary Materials Table S3). The preparation of the guidelines was preceded by a literature search on integrated care concepts [32] in order to formulate and collect questions [33].

### 2.4. Data Collection

Data collection took place from May 2018 to January 2019 in a conference room at a hotel (first focus group and workshop) and at Dresden University Hospital (second focus group). The semi-structured interviews were conducted in the buildings of the health insurance companies and at Dresden University Hospital. Basic patient and physician demographics were collected via a questionnaire. Since the health insurance company representatives were interviewed as representatives of their organization, not as private individuals, no demographic information was requested. Therefore, they were asked to answer the guiding questions from the perspective of the health insurance company (Supplementary Materials Table S3). Focus group moderation (J.M., M.B.) and workshop moderation (J.S., F.M.) was provided by a health services researcher and a member of the Skin Cancer Center staff, respectively, to facilitate responding to participants’ responses with steering and follow-up questions. At the physicians’ workshop, the moderating physician from the Skin Cancer Center was not counted among the physicians present (Table 2). Health insurance company representatives were interviewed exclusively by J.M. Following data collection, a postscript was prepared in each case. All study participants consented to have their conversations recorded using a voice recorder.

### 2.5. Data Evaluation

All audio recordings were transcribed verbatim and anonymized. The analysis of the transcripts was based on the qualitative content analysis of Mayring [34]. In orientation to the guiding questions of the moderation and interview guidelines, deductive main categories were formed, based on which a preliminary category system was designed. This was gradually adapted and supplemented by inductively developing further main categories and subcategories based on the transcribed data material. For this purpose, a coding guideline was developed to define the deductively and inductively developed categories. By adding quotes as anchor examples, the coders were able to assign the answers to the defined categories. We then assigned all quotes that fit according to the category definition of the deductively formed category “Optimization potentials in skin cancer care in Eastern Saxony”. In this way, the inductively formed categories were also defined and supplemented with anchor examples. Categories were formed inductively regardless of how often a topic was mentioned by the participants. The aim was to collect all the content and opinions mentioned. Therefore, we did not quantify or weight the categories by relevance. Following Kuckartz [35], all material was analyzed by at least two coders (J.M., M.L., A.H., or L.R.) in order to compare the category assignments and, in case of disagreement, to discuss and, if necessary, revise the category system. The coders had different professional backgrounds. In an iterative process, the extent to which the coded material could be clearly assigned to the defined categories was examined. The categories were then revised, defined, and provided with anchor examples. Data analysis was supported by MAXQDA software (version 12). For publication, the original quotes were professionally translated into English by a translation agency. To ensure comparability across the three stakeholder groups, we asked comparable guiding questions (Supplementary Materials Table S3) and used comparable category definitions in the coding guidelines. As part of the evaluation, we collected the categories found across all groups and tabulated them to identify whether the optimization potential was named across all groups or only by individual groups.

## 3. Results

### 3.1. Sample Description

A total of 19 patients and one relative (as a proxy for one patient) participated in the two focus groups (duration of audio recordings: 122 min and 130 min). In addition, five relatives participated as accompanying persons. For the stakeholder group of patients, a heterogeneous sample was obtained regarding the characteristics age, gender, stage of disease, and size of the place of residence (Table 1). Eight physicians participated in the workshop (duration of audio recording: 137 min). Participating physicians represented dermatologists and oncologists from urban and small-town settings (Table 2). Of the health insurance companies contacted, a total of three statutory health insurance company representatives agreed to participate in a semi-structured interview (duration of audio recordings: 29 min–72 min).

### 3.2. Categories: Optimization Potential in the Care of Patients with Skin Cancer in the Study Region

**Figure 1:**
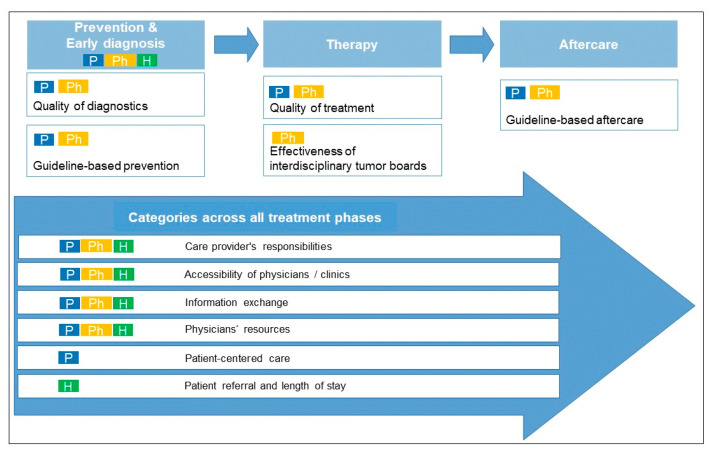
Categories of the needs analysis with assignment to the phases of pretreatment and treatment and the stakeholder groups. P = Patients, Ph = Physicians, H = Health insurance company representatives.

Optimization potential was identified by all three stakeholder groups, while some participants expressed satisfaction with care structures and processes, e.g., because information was provided in a timely manner. In addition, two health insurance company representatives reported that they were only able to assess the cooperation between disciplines, facilities, or help offers to a limited extent or with additional analysis effort. Therefore, the guided question on cooperation was not answered by all health insurance company representatives interviewed. A total of ten categories could be generated from the quotations on optimization potential across all perspectives (Table 3). The categories are assigned to the different phases of care, from prevention and diagnosis to the period of therapy and aftercare (Figure 1). Except for the topic of prevention, these aspects were mainly discussed by patients and physicians. Other categories relate to cross-phase aspects of care that were discussed by all three groups. The statements of the three stakeholder groups are reproduced as paraphrased in the following results.

#### 3.2.1. First Category: Prevention and Early Diagnosis of Skin Cancer

All three stakeholder groups wanted more information and education of the population about skin cancer (e.g., strengthening the awareness of skin cancer as a serious disease). In parallel, patients and health insurance company representatives also discussed skin cancer screening. One patient considered the two-year cycle of screening to be too long. In addition, patients remarked that skin changes were not taken seriously at the beginning or were not recognized as potential skin cancer. Representatives of health insurance companies demanded the general promotion of screening and the lowering of the age limit for the assumption of costs.

#### 3.2.2. Second Category: Patient Referral and Length of Stay

One health insurance representative made the criticism that general practitioners would refer their patients too late to dermatological outpatient practices. This was also associated with the delayed referral to tumor centers. The reasons for this were seen in the competitive situation between the treatment providers and in the budget constraints.

#### 3.2.3. Third Category: Accessibility of Physicians/Clinics

The interviewed physicians sometimes found the accessibility of physicians’ outpatient practices and the clinic difficult. However, some of the patients criticized in particular the local accessibility of medical facilities (e.g., because travel distances are experienced as strenuous in older age groups and in rural areas) and the sometimes difficult access to dermatologists in outpatient practices (e.g., due to long waiting times for an appointment). One health insurance company described the difficult accessibility of physicians’ outpatient practices (e.g., by telephone or due to consultation hours) and the problem of long waiting times for an appointment. A health insurance company representative stated that the accessibility of a dermatological outpatient practice could be a challenge, especially for patients from rural areas. As a result, the physicians’ visit would sometimes not take place.

#### 3.2.4. Fourth Category: Physician Resourcing

There were reports among patients and physicians that indicated a (specialist) physician shortage in Eastern Saxony (e.g., lack of appointments in the outpatient sector). The density of care was also discussed among health insurance company representatives, although there were different perspectives on this. On the one hand, a (specialist) physician shortage was seen (especially for rural regions). On the other hand, the supply density of physicians in eastern Saxony is officially optimal to oversupplied (according to the demand planning guidelines). It was pointed out that neither the accessibility of care services nor mobility and morbidity are taken into account in the official demand planning guidelines, and that these guidelines have been the subject of political discussion for some time with regard to their topicality.

#### 3.2.5. Fifth Category: Care Providers’ Responsibilities

From the patient’s point of view, it was regrettable that an examination that had previously been offered in a hospital close to the patient’s home was later rejected there and the patient was referred to a hospital further away from the patient’s home. One patient described his impression that a hospital does not want to hand over the therapy or examinations to other care services. Patients also criticized the fact that practitioners frequently change or that they lack a fixed contact person. In contrast to the patients, there were statements among the physicians that indicated different expectations regarding the areas of responsibility of care institutions or service providers (e.g., experiences that rehabilitation clinics did not feel responsible for skin cancer patients). With reference to interdisciplinary cooperation, it was described that dermatologists are sometimes not taken seriously by other disciplines. A health insurance company representative described “vanity” between physicians, to the effect that only one’s own opinion is considered the correct one. Treatment pathways with clear guidelines could improve cooperation here. Furthermore, another health insurance company representative doubted whether general practitioners were qualified to perform skin cancer screening is sufficient professionally.

#### 3.2.6. Sixth Category: Quality of Diagnostics and Treatment

Some of the physicians criticized the diagnosis and indication by general practitioners. Patients particularly criticized the quality of treatment by physicians (e.g., not examining the patient or not reading the patient’s file before the appointment) and psychologists (e.g., choosing an inappropriate conversation setting). In Figure 1, this category was mapped twice—in the phase of diagnostics and in the phase of therapy—because quality improvement plays a role at various points in the treatment process.

#### 3.2.7. Seventh Category: Information Exchange

Some participants in all three groups consistently suggested that patient information (e.g., reports of findings) was sometimes not shared with co-/continuing care providers and patients, or not shared in a timely manner. Patients also criticized the way in which findings were communicated (e.g., no feedback in the case of inconspicuous findings).

#### 3.2.8. Eighth Category: Effectiveness of Interdisciplinary Tumor Boards

One physician criticized that interdisciplinary cooperation in the tumor boards is not guaranteed in some cases and that there would be disciplines that stick to their methods. In addition, outdated specialist knowledge and insufficient exchange between the specialist disciplines when presenting patients were criticized.

#### 3.2.9. Nine Category: Guideline-Based Prevention and Aftercare

One relative and one physician criticized the fact that aftercare was sometimes lacking or not carried out at the scheduled times. One physician also reported patient feedback that skin cancer screening by general practitioners did not currently meet official guidelines.

#### 3.2.10. Tenth Category: Patient-Centered Care

Patients criticized that physicians’ letters are sometimes not written in layman’s language, which causes anxiety. In addition, patients would like to receive more information about care services and psychosocial support services, more support and assistance in everyday life (e.g., with self-injections), and a simplification of bureaucratic procedures. Furthermore, an insecurity within the population, as well as the medical staff, towards this potentially fatal disease was mentioned. One patient expressed a desire to reduce uncertainty in dealing with patients.

## 4. Discussion

National and international cancer care goals call for greater integrated oncological care [9,10]. This is particularly important in the interdisciplinary treatment of complex tumor diseases. However, it remains unanswered how these care goals are to be implemented in rural regions. The aim of this study was to explore the perspectives of patients, physicians, and health insurance representatives on regional cooperation in the care of skin cancer patients. Our study region in Saxony, East Germany, contains many examples of regions with an older than average, multimorbid population in rural areas with limited mobility, that are long distances from medical facilities. Although all three stakeholder groups reported good cooperation, they also reported the potential for optimization.

Deficiencies were seen for example in the diffuse responsibilities between care providers. Particularly when different specialists are involved in the provision of care, problems in the transmission of findings, the coordination of appointments, and the treatment processes can impair the quality of care. The results of several studies indicate similar problems to those identified by our participants. The lack of communication and unclear responsibilities between providers can lead to uncertainty and dissatisfaction among patients [36,37,38,39]. One health insurance representative saw delayed referrals as a further problem. However, timely referral to a specialist is imperative to detect a possible disease progression in time and to adequately adapt therapies. In particular, new systemic tumor therapies require continuous monitoring and side effect management. However, “adequate length of stay” is not clearly defined in the official guidelines [40,41]. It is therefore even more important that all those involved in the diagnosis, therapy, and aftercare are informed about the course of the disease as quickly as possible.

In the statements of several participants, discrepancies between their practical experience and the officially communicated specialist density within the study region became clear [42]. On the one hand, some participants mentioned a perceived shortage of physicians, which manifests itself in long waiting times and poor accessibility. On the other hand, health insurance representatives pointed out that the need for outpatient medical care in the region was officially covered [43]. However, the official demand planning does not necessarily reflect the real situation on site. In particular, the treatment of skin cancer patients in an advanced stage results in a considerable additional expenditure, which is expressed in regular examinations, infusions, and aftercare appointments. In addition, there are unscheduled visits to the physician due to the side effects of drug therapies. Therefore, the density of physicians within a region is not the only decisive factor for optimized care, especially in rural regions [44].

This practical experiences described in our analysis support the assumption that rural regions are confronted with various challenges in skin cancer care (including scarce resources with steadily rising skin cancer incidences and long travel distances to care providers). One possible solution could be telemedicine, which is an approach that enables rural coverage and faster information exchange. Telemedical care in rural areas opens up a broad field of new care approaches that cannot be discussed in detail here. For example, telemedicine applications for digital side effect monitoring are currently being implemented [45,46] to support the monitoring of systemic therapies. So far, however, such applications have mainly represented individual regional solutions for specific clinical pictures. A systematic review on the implementation of telemedicine was able to identify various barriers, which vary according to country, organization, and user [47]. Benefits and costs must therefore currently be examined on a case-by-case basis.

Oncological care networks represent another approach to a solution, which aims to improve communication between care providers and provide care close to home [15,17,48]. We believe that networked healthcare, as suggested by Dr. Porter, and his value-based healthcare framework [49] are particularly applicable for cancer prevention and care. Various scenarios are conceivable; for example, one possible focus of the network could be on improving organizational processes between care providers. As suggested in the semi-structured interviews, treatment pathways with defined responsibilities could be used here. However, these were not specified in the interviews. As an example of a care pathway we refer to the network “Cancer Strategic Clinical Network” [15]. Contact databases and overview maps for the medical facilities in the region could also serve as small-scale approaches [50]. However, these treatment pathways must be designed and implemented on a region-specific basis considering the specific local care structure. Furthermore, the guidelines for skin cancer recommend new evidence-based therapies, interdisciplinary therapy, and indications for interdisciplinary tumor boards. Our study should raise awareness and postulate the demand that all patients receive guideline-based therapy regardless of where they live. This demand should be anchored in the guidelines, and Comprehensive Cancer Centers must take responsibility for the region and support the region in realising these guidelines.

### Limitations and Strengths

One strength of our study is that we involved the views of different stakeholder groups and worked through proposals with physicians in a workshop on how stronger networking could be designed in the region. In our experience, physicians are not easy to recruit for such a time-consuming workshop because of their limited time resources. Due to the exploratory study design, a limited number of potential study participants were invited to participate in the study. A larger sample might have helped to identify further categories. At the same time, we found that despite the open-ended questions (Supplementary Materials Table S3), some of the optimization potentials mentioned were repeated already between the focus groups and stakeholder groups (Table 3). It is also possible that people who saw a need for action in skin cancer care were more likely to participate in the surveys, so a selection bias cannot be ruled out. Even though general practitioners were invited to participate in the study, we were not able to recruit them for our workshop. Exploring the perspectives of general practitioners would therefore be relevant in future projects. In terms of data collection, interview and moderation guidelines were used. Due to the chosen methods, effects of social desirability and interviewer bias cannot be excluded. For example, some of the moderators were hospital employees (M.B., F.M). Therefore, it is possible that patients felt particular sympathy or dependency towards the clinic due to their treatment history and therefore withheld criticism. Another limitation is that the data were collected in 2018/2019. In particular, new care problems have arisen in the course of the COVID-19 pandemic. However, it can be assumed that fundamental problems mentioned in the needs assessment still exist or have even worsened, such as communication between the treating physicians. For a more in-depth analysis, it would be desirable to query the needs again; if necessary, by usingva standardized procedure that can also be applied in other regions.

Although the health insurance company representatives were interviewed as representatives of their organization, they also expressed their personal opinions. We have included these in the evaluation in addition to the official positions of the health insurance companies. In the qualitative needs analysis presented here, further questions were also investigated (Supplementary Materials Table S3). Thus, the results presented here represent only a sample of all the analyses. In addition, it must be considered that the statements recorded so far do not allow for quantification. For a comprehensive picture, the additional use of quantitative methods would also be desirable. To our knowledge, this is one of the first studies to investigate the need for a regionally organized cooperated network for skin cancer care. From our point of view, some of our results, such as problems in access to physicians for elderly, multimorbid patients, could also apply to comparable regions with long travel distances to medical care. To determine whether the results can be generalized to other regions and possibly other tumor entities, similar qualitative studies would be interesting. From this, a framework could be derived that targets solutions for care of cancer patients in rural areas.

## 5. Conclusions

In summary, it is clear that a lack of resources is often the cause of the reported problems. These cannot be remedied with stronger networking; however, resources can be pooled and existing structures expanded, which are of direct benefit to medical cooperation and patient care. The assignment of the categories to the individual phases of treatment makes it possible to improve care at this point. For example, following the project, a digital tumor board was initiated at the Skin Cancer Center of the University Hospital Dresden, which enables physicians from different hospitals to present their patients in an easy way to an interdisciplinary team. Furthermore, we established a newsletter on current topics, courses, and workshops, and telephone conferences with physicians to discuss urgent topics directly.

We would be very interested to learn from others who have organized regional healthcare for skin cancer or other dermatological conditions (please email corresponding author) and believe that this is a field with a high potential to attain further benefits to patients’ routine clinical care.

## Figures and Tables

**Table 1:** Characteristics of the patients participating in the focus groups (n = 20 *).

Characteristics		Number
Age	Years: M (SD) ^1^, range	67 (±12.3), 45–83
	Not specified	1
Gender	Female	12
	Male	7
	Not specified	1
Stage of disease (according to AJCC 2017)	Stage I	7
	Stage II	5
	Stage III	4
	Stage IV	4
Size of the place of residence	Large city (≥100,000 inhabitants)	9
	Small-to-medium sized city (≥5000 inhabitants)	7
	Rural community (<5000 inhabitants)	3
	Not specified	1

^1^ M = mean, SD = standard deviation. * = including one relative (as a proxy for one patient).

**Table 2:** Characteristics of physicians participating in the workshop (n = 8).

Characteristics		Number
Age	Years: M (SD) ^1^, range	49 (±8.6), 38–62
	Not specified	1
Gender	Female	6
	Male	2
Specialist discipline	Dermatologists in outpatient practices	4
	Oncologists in outpatient practices	2
	Clinicians from the field of dermatooncology	2
Places of practice	Large city (≥100,000 inhabitants)	2
	Small-to-medium sized city (≥5000 inhabitants)	5
	Not specified	1

^1^ M = mean, SD = standard deviation.

**Table 3:** Overview of the categories generated for the optimization potential in the care of patients with skin cancer in the study region.

Category	Stakeholder Group	Sample Quotes
Prevention and early diagnosis of skin cancer	Patients	“They always have the pictures that look ghastly there and then say, yeah, well if I had something like that I’d go to the physician. You’d have to really show first, like I had this little spot now, that that could be a skin cancer.”
	Physicians	“And skin tumor […], it sounds so harmless. It’s just not colon cancer. Yes, that’s where some of the other specialties are much further ahead of us in enlightenment.”
	Health insurance company representatives	“What I’ve already said, prevention I think needs to be improved generally in mainstream care. For example […] that the age limit must be set lower. […] Payers need to educate patients more. Conversely, of course, the physicians’ outpatient practices, which must also better educate patients. Not only if [a patient] happens to arrive there, but you can inform patients via email, regular mail, saying: attention, it’s time now, you can stop by and do skin cancer screening.”
Patient referral and length of stay	Health insurance company representatives	“The problem I see is mainly in the collaboration in outpatient practice between the GPs and the dermatologists, because the problem is that patients who visit the GP are referred too late. Or in the context of […] the budget problem, too long in the physician’s outpatient practice of the general practitioner, because the general practitioner says, that’s my patient, I don’t want to give him away.”
Accessibility of physicians/clinics	Patients	“[…] so I’m going to be 84 soon now—for me, the drive to [name of a city] is very exhausting. […] I was satisfied, but I can’t do it by myself anymore with trams, buses and everything.”
	Physicians	“But she [a clinic employee] is not always available, so if you have someone [a patient] where you now say that you would like to place them in the clinic quickly. And that’s always the difficult part, to reach the [employee].”
	Health insurance company representatives	“There’s been a lot of problems with visiting the outpatient practice. First of all to reach the practice at all, no matter how, telephone or online appointments, homepage, that is all such a problem. Secondly, when do you get an appointment both with the general practitioner, especially with the dermatologist. […] Because if I have to wait a long time to even reach someone, then get another appointment and wait three months for it, then in case of doubt I don’t even go.”
Physician resourcing	Patients	“So in [place name], there’s only one dermatologist left. I’m already looking for a new dermatologist in my new neighborhood. That’s practically impossible. My dermatologist, she’s retiring in a few years too. At that point, I don’t even know where I’m going to go then, and you’re talking about a network here.”
	Physicians	“And yes, unfortunately, of course, one thing you hear a lot is you can’t get appointments with the local practitioners.”
	Health insurance company representatives	“So there is insufficient coverage here in Saxony, it has to be said. But the problem is getting physicians there. So they’re just not there. […] Infrastructure is the be-all and end-all. And the infrastructure in these village areas is unfortunately just not what it should be.”
Care providers’ responsibilities	Patients	“[…] after the other clinic […] no longer guaranteed me this outpatient examination—That was then the [physician] who referred me to the [name of a clinic] and who said: ‘You’re in good hands there.’ But how I get there and all, of course they don’t care.”
	Physicians	“However, I have to say, I’m always a big fan of the GP-centered care principle, […] we get ten euro for the patient per quarter, the GP gets up to 85. He’s allowed to do a little bit for it. Because that is very often the case that they say […] I’ll send it to you right away, you do it and you also know what you have to do for the lab, I have no idea. We’re lucky sometimes […] that we […] divide up […].”
	Health insurance company representatives	“[…] the vanity between participating care providers must disappear. It’s not that what I say is right, it’s […] treatment pathways. They pretend, the GP can’t say I’m right and you’re not right or the dermatologist says you’re not right. But that this collaboration track can be improved there.”
Quality of diagnostics and treatment	Patients	“I had a different process now in that respect. I had a GP, she was always looking at the files or the computer, but basically never examined me. And that was too much for me at some point. And then I talked to my wife and she put me with another physician. And he asked during the first examination, what kind of spot do you have back here. And that’s when I said I wouldn’t look at myself in the mirror in the back. And that’s when he immediately referred me to the dermatologist. […] That is, if I hadn’t changed GPs and that’s always difficult too, getting a new one.”
	Physicians	“[…] whether we dermatologists have done ourselves such a favor by including general practitioners in the skin check, because […] I also get diagnoses on referral slips. […] So with diagnoses you’ve never heard before.”
Information exchange	Patients	“And to top it all off, yes, I went back to my dermatologist in [place name] several weeks later. Then I thought, well, now my physician will have all the findings here and will tell me something about it again. There was nothing there. She didn’t even know that.”
	Physicians	“[…] A patient who has been cared for there for years and I don’t really know what the status is. […]“
	Health insurance company representatives	“[…] The classic is, of course, discharge management after hospital treatment. In practice, this is a problem that we hear time and time again. Data is submitted late or not at all and then the physician is running after the physician’s letters […].”
Effectiveness of interdisciplinary tumor boards	Physicians	“Simply because so much is changing so quickly in our field that many other disciplines sometimes still [have] views from X years ago […]” “But also this presenting in the tumor board, so unfortunately I’ve seen some tumor boards where it’s really presenting where the dermatologist says I have a patient. Melanoma at that and that stage. I’d like to do that and that right now. And then everyone looks at the wall, yeah well next patient […].”
Guideline-based prevention and aftercare	Patients	“What bothered me afterwards, though, was that there was essentially no aftercare. Further metastases then occurred, so the woman had to have surgery in [month name and year]. And only then was therapy initiated. Why? The question comes to me. Why?”
	Physicians	“I think even with guideline-based aftercare, there are already some issues like that with outpatient-based colleagues. For example, when I look at squamous cell carcinoma, which is not melanoma, but theoretically one would have to do an ultrasound every quarter. For example, that doesn’t really work out either because they don’t really have the facilities.” “And we have the ones where the patients credibly assure us, well I didn’t have to take my clothes off there for a skin check. […] the physician says, he always makes the cross in the computer, that he has done it and if something is wrong, I should come to you in such and such a way. […] the general practitioner, at least in our regions, is not a really reliable entity.”
Patient-centered care	Patients	“The diagnosis really shocks you, doesn’t it? […] No one can tell you what will happen next. And then came all the formalities and going here and going there and stuff. So that was soon worse.” “[…] it’s hard to manage everything. And all the files you get […] and all the appointments. It’s a burden to me. And I haven’t even thought about psychosocial treatment. I didn’t ask for it. No one came to help me there, either.”

## Data Availability

Data presented in this study are available on request from the corresponding author.

## Supplementary Materials

The following supporting information can be downloaded at: https://www.mdpi.com/article/10.3390/curroncol29040212/s1. Table S1: COREQ checklist, Table S2: CASP checklist, Table S3: Guiding questions for the workshop, focus groups and interviews.

## References

[1] Garbe C., Keim U., Eigentler T.K., Amaral T., Katalinic A., Holleczek B., Martus P., Leiter U. (2019). Time trends in incidence and mortality of cutaneous melanoma in Germany. J. Eur. Acad. Dermatol. Venereol..

[2] World Health Organization Cancer. https://www.who.int/news-room/fact-sheets/detail/cancer.

[3] Zentrum für Krebsregisterdaten Krebs in Deutschland 2015/2016. https://www.krebsdaten.de/Krebs/DE/Content/Publikationen/Krebs_in_Deutschland/kid_2019/krebs_in_deutschland_2019.pdf?__blob=publicationFile.

[4] Friedrich S., Kraywinkel K. (2018). Faktenblatt: Epidemiologie des malignen Melanoms in Deutschland. Onkologe.

[5] Larkin J., Chiarion-Sileni V., Gonzalez R., Grob J.-J., Rutkowski P., Lao C.D., Cowey C.L., Schadendorf D., Wagstaff J., Dummer R. (2019). Five-Year Survival with Combined Nivolumab and Ipilimumab in Advanced Melanoma. N. Engl. J. Med..

[6] Robert C., Grob J.J., Stroyakovskiy D., Karaszewska B., Hauschild A., Levchenko E., Chiarion Sileni V., Schachter J., Garbe C., Bondarenko I. (2019). Five-Year Outcomes with Dabrafenib plus Trametinib in Metastatic Melanoma. N. Engl. J. Med..

[7] Ugurel S., Röhmel J., Ascierto P.A., Flaherty K.T., Grob J.J., Hauschild A., Larkin J., Long G.V., Lorigan P., McArthur G.A. (2017). Survival of patients with advanced metastatic melanoma: The impact of novel therapies-update 2017. Eur. J. Cancer.

[8] Wolchok J.D., Chiarion-Sileni V., Gonzalez R., Rutkowski P., Grob J.-J., Cowey C.L., Lao C.D., Wagstaff J., Schadendorf D., Ferrucci P.F. (2017). Overall Survival with Combined Nivolumab and Ipilimumab in Advanced Melanoma. N. Engl. J. Med..

[9] Bundesministerium für Gesundheit Der nationale Krebsplan Stellt Sich vor. https://www.bundesgesundheitsministerium.de/themen/praevention/nationaler-krebsplan/der-nationale-krebsplan-stellt-sich-vor.html.

[10] Cancer Control Joint Action European Guide on Quality Improvement in Comprehensive Cancer Control. https://cancercontrol.eu/archived/uploads/images/Guide/pdf/CanCon_Guide_FINAL_Web.pdf.

[11] Schoffer O., Schülein S., Arand G., Arnholdt H., Baaske D., Bargou R.C., Becker N., Beckmann M.W., Bodack Y., Böhme B. (2016). Tumour stage distribution and survival of malignant melanoma in Germany 2002–2011. BMC Cancer.

[12] Hellmund P., Schmitt J., Roessler M., Meier F., Schoffer O. (2020). Targeted and Checkpoint Inhibitor Therapy of Metastatic Malignant Melanoma in Germany, 2000–2016. Cancers (Basel).

[13] Bundesärztekammer Prozessverbesserung in der Patientenversorgung Durch Kooperation und Koordination Zwischen den Gesundheitsberufen. http://www.bundesaerztekammer.de/fileadmin/user_upload/downloads/FachberufeProzessverbesserung.pdf.

[14] Braun B., Marstedt G., Sievers C. (2011). Zur Bedeutung von Schnittstellen und Übergängen im Deutschen Gesundheitssystem.

[15] Bond T.R., Estey A., Elwi A. (2019). Cancer Strategic Clinical Network: Improving cancer care in Alberta. CMAJ.

[16] Melanoma Network of New Zealand About MelNet. https://www.melnet.org.nz/index.php?p=about.

[17] Comprehensive Cancer Center Ostbayern Regionales Netzwerk. https://www.ccco.de/regionales-netzwerk/.

[18] Kaiser F., Vehling-Kaiser U., Flieser-Hartl M., Weiglein T. (2014). Das Onkologische und Palliativmedizinische Netzwerk Landshut: Lösungsansatz für die zukünftige ambulante und stationäre Versorgung von onkologischen und palliativmedizinischen Patienten in strukturschwachen ländlichen Gebieten. MMW Fortschr. Med..

[19] Müller H.L., Blanke J.-G., Bonse B., Bosse H., Erkel J., Gitmans R., Kolb R., Krull F., Langlitz J., Liebner T. (2010). Verbund PädOnko Weser-Ems--Regionale ambulante Versorgung pädiatrisch-onkologischer Patienten aus der Weser-Ems-Region im Rahmen einer Integrierten Versorgung. Klin. Padiatr..

[20] Landeshauptstadt Dresden Bevölkerungsbestand. https://www.dresden.de/de/leben/stadtportrait/statistik/bevoelkerung-gebiet/Bevoelkerungsbestand.php.

[21] Klinische Krebsregister Sachsen Jahresbericht der Klinischen Krebsregister in Sachsen 2010–2019. https://www.krebsregister-sachsen.de/fileadmin/user_upload/dokumente/auswertungen/Jahresbericht_KKR_Sachsen_2021.pdf.

[22] Demografieportal Regionale Alterung. https://www.demografie-portal.de/DE/Fakten/aeltere-bevoelkerung-regional.html?nn=580048.

[23] Uniklinikum Dresden Hauttumorzentrum am Nationalen Centrum für Tumorerkrankungen Dresden (NCT/UCC). https://www.uniklinikum-dresden.de/de/das-klinikum/universitaetscentren/uhtc.

[24] Städtisches Klinikum Dresden Hautkrebszentrum. https://www.klinikum-dresden.de/hz_khdf/#navigation.

[25] Greenbaum T. (1998). The Handbook for Focus Group Research.

[26] Pelz C., Schmitt A., Meis M. (2004). Knowledge Mapping als Methode zur Auswertung und Ergebnispräsentation von Fokusgruppen in der Markt- und Evaluationsforschung. Forum Qual. Soz./Forum Qual. Soc. Res..

[27] Tong A., Sainsbury P., Craig J. (2007). Consolidated criteria for reporting qualitative research (COREQ): A 32-item checklist for interviews and focus groups. Int. J. Qual. Health Care.

[28] Critical Appraisal Skills Programme (2018). CASP Qualitative Studies Checklist. https://casp-uk.net/casp-tools-checklists.

[29] Patton M.Q. (2015). Qualitative Research & Evaluation Methods: Integrating Theory and Practice.

[30] Misoch S. (2015). Qualitative Interviews.

[31] Bundesinstitut Für Bau-, Stadt- und Raumforschung (BBSR) Laufende Stadtbeobachtung–Raumabgrenzungen. https://www.bbsr.bund.de/BBSR/DE/forschung/raumbeobachtung/Raumabgrenzungen/deutschland/gemeinden/StadtGemeindetyp/StadtGemeindetyp.html.

[32] Meier U., Diener H.C. (2007). Kommission Integrierte Versorgung der Deutschen Gesellschaft für Neurologie (Ed.) Integrierte Versorgung in der Neurologie: Integrierte Versorgungskonzepte und Kooperative Versorgungsstrukturen.

[33] Helfferich C. (2011). Die Qualität Qualitativer Daten: Manual für Die Durchführung Qualitativer Interviews.

[34] Mayring P. (2010). Qualitative Inhaltsanalyse: Grundlagen und Techniken.

[35] Kuckartz U. (2016). Qualitative Inhaltsanalyse: Methoden, Praxis, Computerunterstützung.

[36] Lang C., Gottschall M., Sauer M., Köberlein-Neu J., Bergmann A., Voigt K. (2019). “Da kann man sich ja totklingeln, geht ja keiner ran”–Schnittstellenprobleme zwischen stationärer, hausärztlicher und ambulant-fachspezialisierter Patientenversorgung aus Sicht Dresdner Hausärzte. Gesundheitswesen.

[37] Easley J., Miedema B., O’Brien M.A., Carroll J., Manca D., Webster F., Grunfeld E. (2017). The role of family physicians in cancer care: Perspectives of primary and specialty care providers. Curr. Oncol..

[38] Walsh J., Harrison J.D., Young J.M., Butow P.N., Solomon M.J., Masya L. (2010). What are the current barriers to effective cancer care coordination? A qualitative study. BMC Health Serv. Res..

[39] Dossett L.A., Hudson J.N., Morris A.M., Lee M.C., Roetzheim R.G., Fetters M.D., Quinn G.P. (2017). The primary care provider (PCP)-cancer specialist relationship: A systematic review and mixed-methods meta-synthesis. CA Cancer J. Clin..

[40] Leitlinienprogramm Onkologie (Deutsche Krebsgesellschaft, Deutsche Krebshilfe, AWMF) Diagnostik, Therapie und Nachsorge des Melanoms: Langversion 3.3, 2020, AWMF Registernummer: 032/024OL. https://www.leitlinienprogramm-onkologie.de/fileadmin/user_upload/Downloads/Leitlinien/Melanom/Melanom_Version_3/LL_Melanom_Langversion_3.3.pdf.

[41] Leitlinienprogramm Onkologie (Deutsche Krebsgesellschaft, Deutsche Krebshilfe, AWMF) Diagnostik, Therapie und Nachsorge des Melanoms: Leitlinienreport, Version 3.2., 2019, AWMF Registernummer: 032/024OL. https://www.leitlinienprogramm-onkologie.de/fileadmin/user_upload/Downloads/Leitlinien/Melanom/Melanom_Version_3/LL_Melanom_Leitlinienreport_3.2.pdf.

[42] Gemeinsamer Bundesausschuss Bedarfsplanungs-Richtlinie. https://www.g-ba.de/richtlinien/4/.

[43] Kassenärztliche Vereinigung Sachsen. Bedarfsplan 2020: Anlage 1.1.5.: Planungsblatt Hautärzte, 2020. https://www.kvsachsen.de/fileadmin/data/kvs/img/Aktuelles/Der_Weg_in_die_Praxis/200129_Bedarfsplan_2020_Stand_20200127_final_oeff.pdf.

[44] Hüther M., Südekum J., Voigtländer M. (2019). Die Zukunft der Regionen in Deutschland: Zwischen Vielfalt und Gleichwertigkeit.

[45] Jünger M., Arnold A., Lutze S. (2019). Teledermatologie zur notfallmedizinischen Patientenversorgung: Zweijahreserfahrungen mit teledermatologischer Notfallversorgung. Hautarzt.

[46] Mohr P., Tadmouri A., Suissa J., Alivon M., Meyer N. (2020). 1140P A digital companion for patients with BRAF-mutant advanced melanoma treated with targeted therapies: TAVIE skin app. Ann. Oncol..

[47] Kruse C.S., Karem P., Shifflett K., Vegi L., Ravi K., Brooks M. (2018). Evaluating barriers to adopting telemedicine worldwide: A systematic review. J. Telemed. Telecare.

[48] Melanoma Network of New Zealand MelNet Strategic Plan. https://www.melnet.org.nz/uploads/Strategic-Plan.pdf.

[49] Porter M.E. (2010). What is value in health care?. N. Engl. J. Med..

[50] Herbst F.A., Heckel M., Stiel S., Ostgathe C. (2017). Kompetent vernetzt–optimal versorgt!: Förderliche Faktoren der Zusammenarbeit in hospizlich-palliativen Versorgungsnetzwerken in Bayern. Bundesgesundheitsblatt Gesundh. Gesundh..

