# Supplementary material for: A Qualitative Needs Analysis of Skin Cancer Care from the Perspectives of Patients, Physicians, and Health Insurance Representatives—A Case Study from Eastern Saxony, Germany
Source: Curr Oncol. 2022 Apr 9;29(4):2583–98. doi: 10.3390/curroncol29040212 (PMC9029997; doi:10.3390/curroncol29040212)
Supplement: Supplementary file 1 [file curroncol-29-00212-s001.zip › Table S2 CASP checklist.pdf]

Article: "A Qualitative Needs Analysis of Skin Cancer Care from the Perspectives of Patients, Physicians and Health Insurance Representatives – A Case Study from Eastern Saxony, Germany"

Supplementary Materials

Table S2. CASP checklist. (Critical Appraisal Skills Programme (2018). CASP Qualitative Studies Checklist. Available online: https://casp-uk.net/casp-tools-checklists (accessed on 24 March 2022)).

Question	Rating	Comments
Was there a clear statement of the aims of the research?	Yes	• What was the goal of the research: Reported on pages 1–2<br>• Why it was thought important its relevance: Reported on pages 1-2
Is a qualitative methodology appropriate?	Yes	• If the research seeks to interpret or illuminate the actions and/or subjective experiences of research participants: Reported on page 2<br>• Is qualitative research the right methodology for addressing the research goal: Reported on pages 2 and 12
Was the research design appropriate to address the aims of the research?	Yes	• If the researcher has justified the research design (e.g. have they discussed how they decided which method to use): Reported on page 3
Was the recruitment strategy appropriate to the aims of the research?	Yes	• If the researcher has explained how the participants were selected: Reported on pages 3-4<br>• If they explained why the participants they selected were the most appropriate to provide access to the type of knowledge sought by the study: Reported on pages 1-2<br>• If there are any discussions around recruitment (e.g. why some people chose not to take part): Reported on page 12
Was the data collected in a way that addressed the research issue?	Yes	• If the setting for the data collection was justified: Reported on pages 2-3 and 10<br>• If it is clear how data were collected (e.g. focus group, semi-structured interview etc.); If the researcher has justified the methods hosen: Reported on pages 3-4<br>• If the researcher has made the methods explicit (e.g. for interview method, is there an indication of how interviews are conducted, or did they use a topic guide): Reported on pages 4-5<br>• If methods were modified during the study. If so, has the researcher explained how and why: N/A<br>• If the form of data is clear (e.g. tape recordings, video material, notes etc.): Reported on pages 4-5<br>• If the researcher has discussed saturation of data: Reported on page 12
Has the relationship between researcher and participants been adequately considered?	Yes	• If the researcher critically examined their own role, potential bias and influence during (a) formulation of the research questions (b) data collection, including sample recruitment and choice of location: Reported on page 12<br>• How the researcher responded to events during the study and whether they considered the implications of any changes in the research design: N/A
Have ethical issues been taken into consideration?	Yes	• If there are sufficient details of how the research was explained to participants for the reader to assess whether ethical standards were maintained; If the researcher has discussed issues raised by the study (e.g. issues around informed consent or confidentiality or how they have handled the effects of the study on the participants during and after the study): Reported on page 13<br>• If approval has been sought from the ethics committee: Reported on page 13

Was the data analysis sufficiently rigorous?	Yes	• If there is an in-depth description of the analysis process: Reported on page 5<br>• If thematic analysis is used. If so, is it clear how the categories/themes were derived from the data: Reported on page 5<br>• Whether the researcher explains how the data presented were selected from the original sample to demonstrate the analysis process: Reported on page 5<br>• If sufficient data are presented to support the findings: Reported on pages 5-10<br>• To what extent contradictory data are taken into account: Reported on pages 6-7<br>• Whether the researcher critically examined their own role, potential bias and influence during analysis and selection of data for presentation: Reported on page 5
Is there a clear statement of findings?	Yes	• If the findings are explicit; If there is adequate discussion of the evidence both for and against the researcher's arguments: Reported on pages 10-12<br>• If the researcher has discussed the credibility of their findings (e.g. triangulation, respondent validation, more than one analyst): Reported on page 5<br>• If the findings are discussed in relation to the original research question: Reported on pages 10-13
How valuable is the research?	average	• If the researcher discusses the contribution the study makes to existing knowledge or understanding (e.g. do they consider the findings in relation to current practice or policy, or relevant research-based literature: Reported on pages 10-12<br>• If they identify new areas where research is necessary; If the researchers have discussed whether or how the findings can be transferred to other populations or considered other ways the research may be used: Reported on pages 10-13
