# Supplementary material for: A Qualitative Needs Analysis of Skin Cancer Care from the Perspectives of Patients, Physicians, and Health Insurance Representatives—A Case Study from Eastern Saxony, Germany
Source: Curr Oncol. 2022 Apr 9;29(4):2583–98. doi: 10.3390/curroncol29040212 (PMC9029997; doi:10.3390/curroncol29040212)
Supplement: Supplementary file 1 [file curroncol-29-00212-s001.zip › Table S3 Guiding questions for the workshop, focus groups and interviews.pdf]

**Article: "A Qualitative Needs Analysis of Skin Cancer Care from the Perspectives of Patients, Physicians and Health Insurance Representatives – A Case Study from Eastern Saxony, Germany"**

**Supplementary Materials**

**Table S3.** Guiding questions for the workshop, focus groups and interviews.

| Guiding questions for the workshop with physicians |
|---|

- Prevention, treatment, and aftercare of skin cancer may involve multiple disciplines, facilities and/or help offers. As a physician, how would you rate the cooperation between these parties?
- Do you see potential for optimisation in the cooperation between the disciplines, facilities and/or help offers?
- As a physician, what goal(s) do you see for the network and how can their success be measured?
- Who/which disciplines, facilities and/or help offers should be involved in the network?
- What services/activities would the individual disciplines, facilities and/or help offers must provide in order to achieve the network's goals?
- As a physician, do you see advantages in the network?
- As a physician, do you see any disadvantages and/or barriers in the network?
- What should the exchange of information in the network look like for you as a physician? Please give reasons for your view. As a physician, what is your view of an electronic case file in the network?

| Guiding questions for the focus groups with patients and relatives |
|---|

- Prevention, treatment, and aftercare of the malign melanoma may involve multiple disciplines, facilities and/or help offers. As a patient/relative, how would you rate the cooperation between these parties?
- Do you see potential for optimisation in the cooperation between the disciplines, facilities and/or help offers? What has been your experience as a patient/relative?
- Based on your answers, do you see a need for a network in which several disciplines, facilities and/or help offers work together?
- As a patient/relative, what goal(s) do you see for the network? How would you know that this goal/these goals have been met?
- Who/which disciplines, facilities and/or help offers should be involved in the network?
- What services/activities would the individual disciplines, facilities and/or help offers must provide in order to achieve the network's goals?
- As a patients/relative, do you see advantages in the network?
- As a patient/relative, do you see any disadvantages and/or barriers in the network?
- What should the exchange of information in the network look like for you as a patient/relative? As a patient/relative, what is your view of an electronic case file in the network?

| Guiding questions for the interviews with health insurance company representatives |
|---|

- Prevention, treatment, and aftercare of the malign melanoma may involve multiple disciplines, facilities and/or help offers. From the perspective of the health insurance company, how would you rate the cooperation between these parties?
- Do you see potential for optimisation in the cooperation between the disciplines, facilities and/or help offers? What has been your experience from the perspective of the health insurance company?
- Do you see a need for a network in which several disciplines, facilities and/or help offers work together?
- From the perspective of the health insurance company, what goal(s) do you see for the network? How would you know that this goal/these goals has/have been met?
- Who/which disciplines, facilities and/or help offers should be involved in the network? What services/activities would the individual disciplines, facilities and/or help offers must provide in order to achieve the network's goals?
- How would you see the role of the health insurance company in the network?
- What would a network have to include for you to participate from a health insurance perspective?
- In your opinion, what criteria would a network have to fulfill in order for you, as a representative of the health insurance company, to consider financial support?
- From the perspective of the health insurance company, do you see advantages in the network?
- From the perspective of the health insurance company, do you see any disadvantages and/or barriers in the network?
- What should the exchange of information in the network look like for you as a health insurance company? From the perspective of the health insurance company, what is your view of an electronic case file in the network?
